# Antibiotic Susceptibility Report of Bacteria Isolated From Blood Cultures of Neutropenic Sepsis Patients Undergoing Chemotherapy

**DOI:** 10.7759/cureus.85903

**Published:** 2025-06-13

**Authors:** Tajudeen Musbau, Pyae Phyo Thinn, Aye Moh Moh Paing, Thet Htar Swe, Namitha Sebastian, Eiei Phyo, Bahaa Al-Bubseeree, David Griffith

**Affiliations:** 1 Hematology, Victoria Hospital, Kirkcaldy, Kirkcaldy, GBR; 2 Medicine, Victoria Hospital, Kirkcaldy, Kirkcaldy, GBR; 3 Internal Medicine, NHS Fife, Scotland, GBR; 4 Microbiology, Victoria Hospital, Kirkcaldy, Kirkcaldy, GBR

**Keywords:** acute myeloid leukemia (aml), antibiotics, bacteria, cvc, e. coli, gram negative, gram positive, neutropenic sepsis, sensitivity

## Abstract

Background

Febrile neutropenia often occurs as a serious complication in patients receiving chemotherapy. It is regarded as an oncologic emergency due to its high morbidity and mortality, necessitating the prompt initiation of empirical antibiotic therapy. While gram-negative bacteria have historically been the primary cause of bloodstream infections in febrile neutropenic patients, recent shifts in bacterial epidemiology and antimicrobial resistance patterns underscore the need for regular evaluation of local data to inform treatment strategies.

Methods

This retrospective cohort study was conducted in the Department of Hematology at Victoria Hospital, Kirkcaldy, from January to December 2024. It analyzed blood culture results, antibiotic use, chemotherapy regimens, patient characteristics, and microbial susceptibility patterns. A total of 63 patients with an absolute neutrophil count of less than 0.5 were selected through a simple convenience sampling method. Demographic data were collected from ward admission records using a standardized data collection sheet. Blood culture results were retrieved from the laboratory information management system and analyzed using IBM SPSS Statistics for Windows, Version 23.0 (Released 2015; IBM Corp., Armonk, NY, USA).

Results

Among the 63 patients, 20 (32%) had at least one positive blood culture, with a male-to-female ratio of 3:2. Gram-positive bacteria accounted for the majority of isolates (11; 55%), followed by gram-negative bacteria (eight; 40%), and mixed growth in one case (5%). *Escherichia coli* was the most common isolate (five; 25%), followed by *Klebsiella* species and *Streptococcus viridans* (four; 20% each). *Staphylococcus epidermidis* was identified in 3 cases (15%). The most common pathogens associated with line infections were *S. epidermidis* (three; 25%), *S. viridans* (three; 25%), and *Klebsiella* species (four; 33%). A statistically significant association was observed between the presence of a central venous line and the occurrence of bacteremia (p < 0.05). Most gram-negative isolates were susceptible to beta-lactam antibiotics and gentamicin, while gram-positive organisms showed high sensitivity to vancomycin and linezolid. Although gram-positive bacteria were the most frequently isolated organisms overall, *E. coli* was the single most common isolate and demonstrated susceptibility to the current first- and second-line antibiotics used in the treatment of neutropenic sepsis.

Conclusions

The findings of this study are broadly consistent with existing literature on bloodstream infections in neutropenic cancer patients, particularly in terms of the microbial spectrum and the association with central venous lines. However, variations in pathogen prevalence and antibiotic susceptibility may reflect local epidemiology or specific cohort characteristics. These results highlight the need to continually assess and update antibiotic guidelines to mitigate the development of drug-resistant strains, ensure adherence to current protocols, and improve the accuracy of empirical antibiotic prescribing.

## Introduction

Neutropenic sepsis is a potentially life-threatening complication of neutropenia. It is defined as a temperature exceeding 38°C or the presence of any signs and/or symptoms of sepsis in a patient with an absolute neutrophil count (ANC) of 0.5 × 10⁹/L or lower [1]. The Scottish Antimicrobial Prescribing Group expands this definition to include patients with a neutrophil count below 1.0 × 10⁹/L within 21 days of receiving chemotherapy, accompanied by one or more of the following: fever, hypothermia, systemic inflammatory response syndrome criteria, sepsis, or septic shock [2].

Patients undergoing chemotherapy are particularly vulnerable to neutropenic sepsis, which often originates from infections of the respiratory, gastrointestinal, urinary, or integumentary systems, or from indwelling devices such as Hickman lines. Clinical symptoms may be subtle or atypical, and fever can be absent due to immunosuppression, making early recognition and intervention critical [3]. The incidence of febrile episodes and sepsis during the neutropenic phase following intensive myelosuppressive chemotherapy ranges from 70% to 100%, depending on factors such as the intensity of chemotherapy, the severity and duration of neutropenia, the patient’s overall health status, and previous treatments. Bacteremia occurs in approximately 10-30% of febrile neutropenic episodes, and some studies report that around 40% of patients progress to severe sepsis or septic shock. An overall rise in septic episodes is anticipated [4-8].

Gram-negative bacteria remain the predominant pathogens, with *Klebsiella pneumoniae *and *Escherichia coli *being among the most commonly isolated organisms. These strains may exhibit resistance to piperacillin-tazobactam but generally retain sensitivity to meropenem and amikacin. *Pseudomonas aeruginosa *and *Staphylococcus epidermidis *are also frequently identified. *P. aeruginosa* typically responds to meropenem and amikacin, whereas *S. epidermidis *is usually sensitive to vancomycin and linezolid [1,9-12]. Early initiation of empirical antibiotic therapy, informed by local resistance trends, is vital to improving clinical outcomes.

By examining the prevailing microbiological profiles and resistance patterns, this study seeks to support rational antibiotic use, curb the development of resistant organisms, and enhance adherence to evidence-based treatment guidelines, ultimately aiming to optimize patient care.

## Materials and methods

This retrospective cohort study was conducted in the Department of Hematology at Victoria Hospital, Kirkcaldy, between January and December 2024. It included all blood culture samples taken from patients aged 16 years or older who were admitted to the hematology ward with neutropenic sepsis secondary to hematologic or oncologic malignancies. A total of 63 patients with an ANC of less than 0.5 × 10⁹/L were selected using a simple convenience sampling method.

For blood culture collection, a single set (aerobic and anaerobic) was obtained from patients without a central line, while two sets were collected from those with a Hickman line, PICC line, or portacath. Samples were drawn during temperature spikes of ≥38°C to reduce contamination risk, following local protocol, and were labeled appropriately using Bactec bottles before being sent to the microbiology laboratory for processing.

Samples were incubated at 35°C in the Bactec FX system, an automated instrument that detects microbial growth by monitoring CO₂ production, a metabolic byproduct. When growth was detected, the system flagged the sample as positive. A Gram stain was then performed to classify the bacteria as either gram-positive or gram-negative. Positive cultures were subcultured onto blood and MacConkey agar plates and incubated at 37°C under both aerobic and anaerobic conditions for 24-48 hours.

Bacterial colonies grown from subcultures were identified using matrix-assisted laser desorption/ionization time-of-flight mass spectrometry, a rapid technique that identifies bacteria based on their unique protein fingerprint. Additional biochemical tests were conducted when needed to refine identification. Antibiotic susceptibility testing was performed using the Vitek 2 system, an automated platform that provides minimum inhibitory concentration values along with interpretive categories. Cultures were deemed negative if no growth was detected after five days of incubation, although in special cases, the incubation period was extended.

Patients were eligible for inclusion if they presented with neutropenic sepsis, defined as an ANC of less than 500 cells/mm³, were undergoing chemotherapy at the time of hospital admission, and were over 16 years of age. Patients were excluded if they were 16 years old or younger, had an ANC greater than 500 cells/mm³, or if their neutropenia was due to immunosuppressant medication rather than chemotherapy.

Demographic data were collected from ward admission lists using a standardized data collection worksheet. Microbiological data were extracted from laboratory information management system reports. All data were analyzed using IBM SPSS Statistics for Windows, Version 23.0 (Released 2015; IBM Corp., Armonk, NY, USA). Statistical associations were assessed using the chi-square test, with significance defined as a P-value less than 0.05.

## Results

This study included 63 patients, of whom 32 were male and 31 were female, with a mean age of 64.4 years. The average neutrophil count was 0.22 × 10⁹/L, with the lowest recorded count being 0. The majority of patients, 54 (86%), had hematological cancers or related disorders, while nine patients (14%) were diagnosed with nonhematological malignancies.

**Figure 1:**
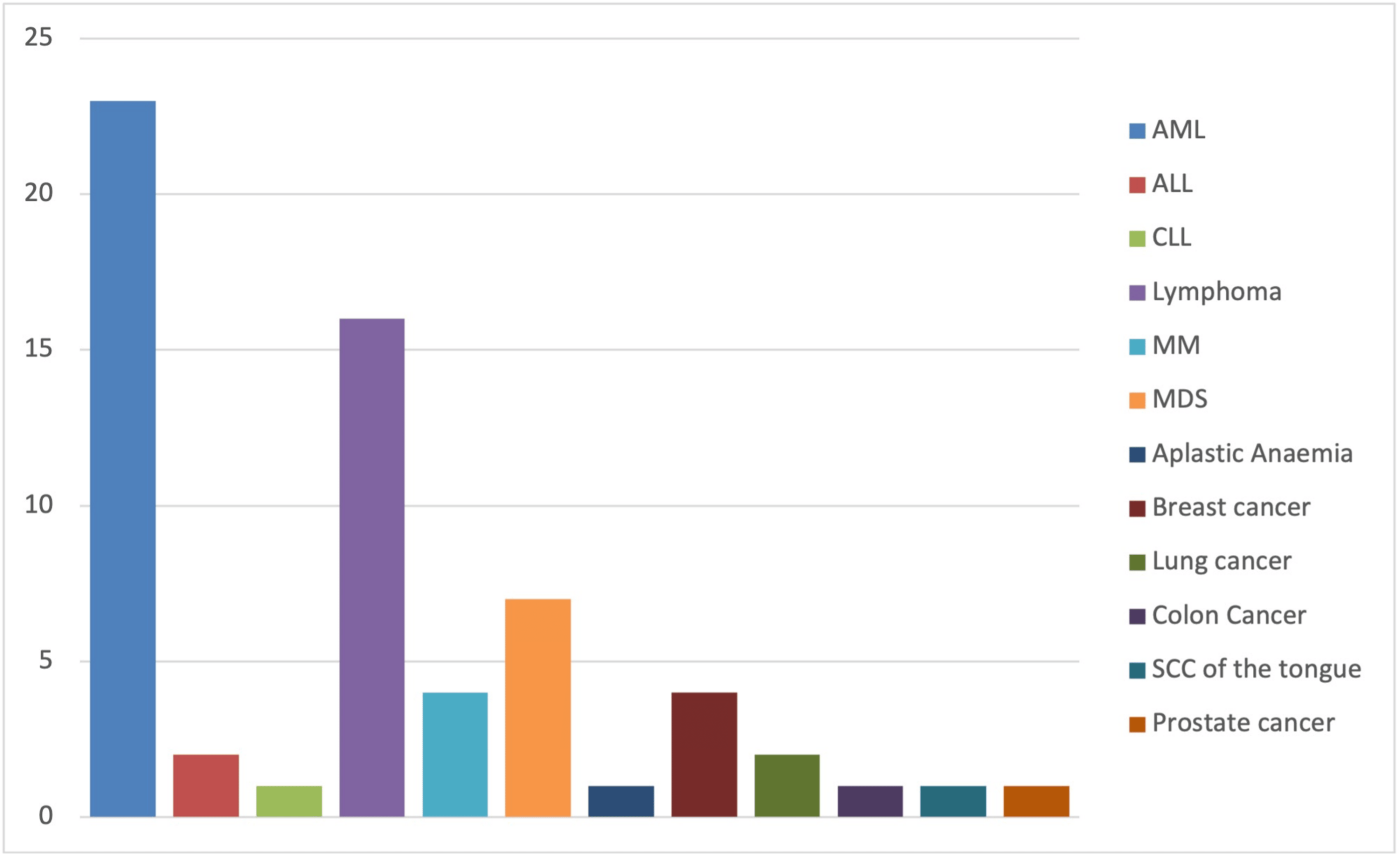
Neutropenic sepsis admissions in hematological versus nonhematological cancer patients within 21 days following chemotherapy administration

Among the hematological cancers, acute myeloid leukemia (AML) was the most prevalent, accounting for 23 cases (36%), followed by lymphoma with 16 cases (25%), myelodysplastic syndrome (MDS) with seven cases (11%), multiple myeloma with four cases (6%), acute lymphoblastic leukemia (ALL) with two cases (3%), chronic lymphocytic leukemia (CLL) with one case (2%), and aplastic anemia (AA) with one case (2%) (Figure 1).

Nonhematological cancers included breast cancer (four cases; 6%), lung cancer (two cases; 3%), colon cancer (one case; 2%), prostate cancer (one case; 2%), and squamous cell carcinoma of the tongue (one case; 2%).

**Figure 2:**
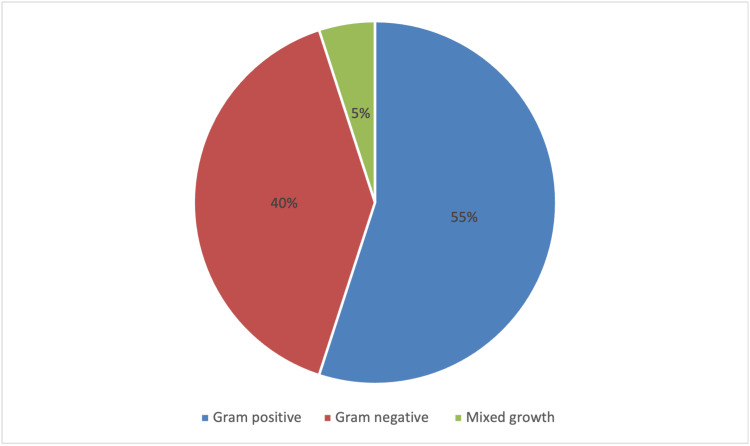
Distribution of patients by type of bacterial infection: gram-positive, gram-negative, and mixed gram-positive/gram-negative growth

Of the 63 patients included in the study, 43 (68%) had no microbial growth on blood cultures. The remaining 20 patients (32%) had at least one positive blood culture result, with a male-to-female ratio of 3:2. Among the positive cultures, 11 (55%) grew gram-positive bacteria, eight (40%) grew gram-negative bacteria, and one (5%) exhibited mixed growth (Figure 2).

**Figure 3:**
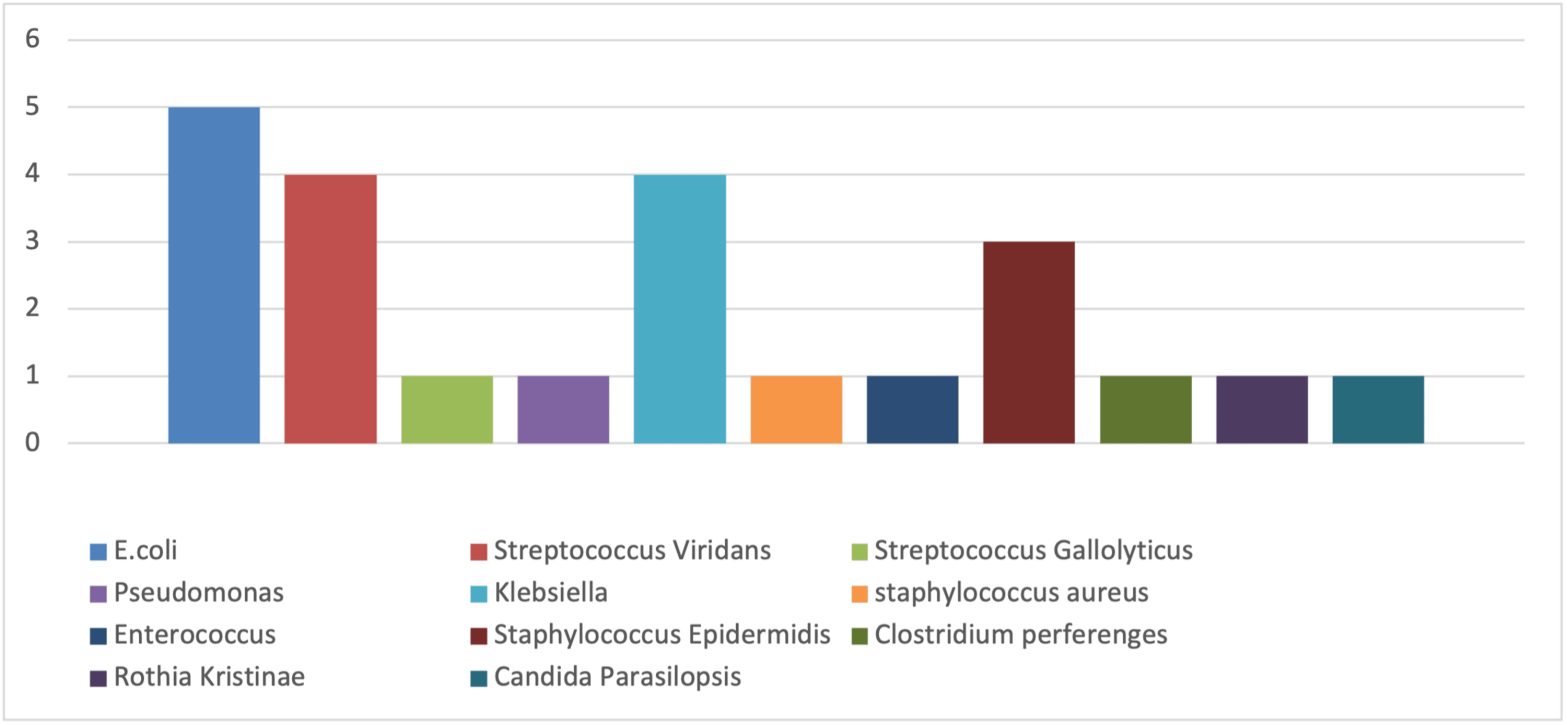
Frequency of individual bacterial isolates identified in clinical specimens from patients diagnosed with neutropenic sepsis The data reflect the distribution of various bacterial species across the study cohort.

*E. coli *was the most frequently isolated organism, accounting for five (25%) of the total isolates, predominantly in female patients (three isolates; 60%). Among the gram-positive isolates, the most common organisms were *Streptococcus viridans *(four isolates; 36%) and *S. epidermidis *(three isolates; 27%). Other gram-positive bacteria identified included *Enterococcus faecium*, *Streptococcus gallolyticus*, *Staphylococcus aureus*, *Clostridium perfringens*, and *Rothia kristinae*, each representing a smaller portion of isolates (Figure 3). 

In the gram-negative group, *E. coli *was the most common pathogen, found in four (55%) of the isolates, followed by *Klebsiella *species (three isolates; 44%) and *P. aeruginosa *(one isolate; 11%). In addition, *Candida parapsilosis *was identified in one blood culture, and another culture showed mixed growth involving *Enterococcus*, *Klebsiella*, and *E. coli*.

Among the 63 patients, 19 (30%) had an indwelling chemotherapy line. These included 13 (68%) Hickman lines, five (26%) peripherally inserted central catheters (PICCs), and one (6%) chemo port-a-cath. Among patients with these lines, 12 (63%) had positive blood cultures. Specifically, nine (75%) of patients with Hickman lines and three (25%) with PICCs had positive cultures. The predominant pathogens associated with line infections were *S. epidermidis *(three isolates; 25%), *S. viridans *(three isolates; 25%), and *Klebsiella *species (four isolates; 33%). A statistically significant association was observed between the presence of a central venous line and bacteremia (p < 0.05, Table 1).

**Table 1:** Statistical association between CVCs and blood culture results based on chi-square analysis ^* ^Statistically significant result ^a^ No cells (0.0%) have an expected count less than 5; the minimum expected count is 6.03. ^b^ Analysis computed only for a 2×2 table p-values are considered statistically significant when p < 0.05. CVC, central venous catheter

Test	Value	df	Asymptotic significance (two-sided)	Exact significance (two-sided)	Exact significance (one-sided)
Pearson chi-square	12.388^a^	1	p < 0.001^*^	-	-
Continuity correction^b^	10.4	1	p = 0.001^*^	-	-
Likelihood ratio	12.01	1	p < 0.001^*^	-	-
Fisher’s exact test	-	-	-	p < 0.001^*^	p < 0.001^*^
Linear-by-linear association	12.192	1	p < 0.001^*^	-	-
Number of valid cases	63	-	-	-	-

Most patients with positive blood cultures were initially treated with a combination of piperacillin and tazobactam, with regimens adjusted according to the antibiotic susceptibility profile of the isolated organisms. All *E. coli *isolates (5; 100%) were fully susceptible to piperacillin-tazobactam, meropenem, and gentamicin. Co-amoxiclav and ampicillin were effective against 80% of the isolates, while only 60% showed sensitivity to ciprofloxacin. Amoxicillin had the lowest effectiveness, with only 20% of isolates being susceptible.

All *Klebsiella *isolates (four; 100%) were sensitive to piperacillin-tazobactam, meropenem, gentamicin, and ciprofloxacin. The single Pseudomonas isolate showed sensitivity to meropenem, gentamicin, and tobramycin (Table 2). *S. epidermidis *and *S. viridans *isolates demonstrated 100% sensitivity to vancomycin and linezolid, with 75% of *S. viridans *isolates also showing susceptibility to benzylpenicillin (Table 3). The chemotherapy regimens most frequently associated with neutropenic sepsis in this cohort were daunorubicin and cytarabine in eight patients (13%), azacytidine alone in eight patients (13%), venetoclax combined with azacytidine in seven patients (11%), and R-CHOP in four patients (6%).

**Table 2:** Antibiotic susceptibility profiles of commonly isolated gram-negative bacteria from blood cultures of patients with neutropenic sepsis in this study A plus sign (+) indicates the number of isolates sensitive to each antibiotic, while a minus sign (-) denotes antibiotics that were not tested against the respective organisms.

Bacteria	Piperacillin-tazobactam	Meropenem	Gentamicin	Co-amoxiclav	Ampicillin	Ciprofloxacin	Amoxicillin	Tobramycin
*Escherichia coli*	+++++	+++++	+++++	++++	++++	+++	+	-
*Klebsiella*	++++	++++	++++	-	-	++++	-	-
*Pseudomonas*	-	+	+	-	-	-	-	+

**Table 3:** Antimicrobial susceptibility patterns of common gram-positive bacteria isolated from blood cultures of patients with neutropenic sepsis in this study

Bacteria	Vancomycin	Teicoplanin	Linezolid	Benzylpenicillin
*Streptococcus viridans*	++++	++++	++++	+++
*Staphylococcus epidermidis*	+++	-	+++	-
*Enterococcus*	+	+	+	-

## Discussion

The study examined a cohort of 63 cancer patients, the majority of whom had hematological malignancies such as AML, lymphoma, MDS, ALL, CLL, AA, and multiple myeloma. It emphasizes the high prevalence of neutropenia (low neutrophil count) in these patients and the resulting risk of bacteremia. We observed that hematological malignancies were more frequently associated with neutropenic illness compared to nonhematological malignancies, with AML being the most common underlying condition. Additionally, 98% of the patients developed neutropenia as a result of chemotherapy. Notably, more positive blood cultures were found in male patients, a finding that contrasts with a study conducted in Pakistan, where a higher incidence was reported among females [13].

In our cohort, the incidence of bacteremia was 32%, aligning with rates reported in other studies involving neutropenic cancer patients, where positivity ranges from 20% to 50%. For example, Khan et al. (2021) found a 22% blood culture positivity rate among neutropenic patients, while Swati et al. reported a rate of 41.2%, both comparable to our results [13,14]. Furthermore, our finding of a 30% positivity rate among patients with indwelling central venous lines highlights a significant association between line presence and bloodstream infections. This observation is consistent with existing literature identifying central venous catheters (CVCs) as major risk factors for infection in immunocompromised individuals. A study by Lee et al. (2018) similarly reported a high infection rate in patients with CVCs, with common pathogens such as *S. epidermidis *and *Klebsiella *species also observed in our study [15]. These findings reinforce the importance of rigorous monitoring and aseptic care for patients with central venous access, particularly those undergoing chemotherapy.

Our results revealed a higher prevalence of gram-positive bacteremia (55%) compared to gram-negative cases (40%). This pattern diverges from what is typically seen in neutropenic sepsis, where gram-negative organisms often predominate. The observed discrepancy may stem from factors such as the widespread use of CVCs, which are more prone to gram-positive colonization, and the routine use of fluoroquinolone prophylaxis in high-income countries, which primarily targets gram-negative bacteria [16]. Despite this, *E. coli *was the most commonly isolated pathogen among febrile neutropenic patients, followed by *Klebsiella *species. This mirrors the findings of Jung et al., who reported *E. coli *as accounting for nearly half of all isolates [17]. Similarly, John et al. identified *Klebsiella *and *E. coli *as leading causes of neutropenic sepsis [18]. Conversely, a study from Pakistan identified *Acinetobacter *as the most frequently isolated organism, while an Indian study found *Klebsiella *to be the most prevalent gram-negative pathogen [13,19]. Coagulase-negative *Staphylococcus *and *Klebsiella *were the most common organisms implicated in line-associated infections, consistent with findings by Sattler et al. [20].

Our antibiotic susceptibility testing showed that *E. coli *exhibited the highest sensitivity to beta-lactam antibiotics. Additionally, *Enterococcus*, coagulase-negative *Staphylococcus*, and *Streptococcus *species demonstrated 100% sensitivity to vancomycin and linezolid, with approximately 67% of *S. epidermidis *isolates also sensitive to benzylpenicillin. These results align with previous studies in Uganda, where no vancomycin resistance was detected among gram-positive isolates [21]. This contrasts with data from other cancer centers, such as those cited by Weinstock et al., which report high rates of vancomycin-resistant *Enterococcus *species [22]. We also found that *Klebsiella *species were sensitive to fluoroquinolones and gentamicin, while *Pseudomonas *showed high susceptibility to meropenem, tobramycin, and gentamicin. These results are consistent with those from a study conducted in Estonia [23].

Limitations

This study was conducted at a single healthcare facility, which may limit the generalizability of the findings to other institutions with different patient populations or antimicrobial stewardship policies. Variations in chemotherapy regimens may have influenced the severity and duration of neutropenia, thereby affecting the risk of infection and the profile of isolated organisms. Furthermore, the study did not assess clinical outcomes such as mortality or treatment success, limiting our ability to correlate susceptibility patterns with clinical effectiveness.

## Conclusions

The findings of this study are broadly consistent with the existing literature on bloodstream infections in neutropenic cancer patients, particularly regarding the microbial profile and the significant role of CVCs in infection risk. However, some variation in pathogen prevalence and antibiotic resistance patterns may reflect local microbiological trends or unique cohort characteristics. According to our hospital protocol, empirical treatment for neutropenic fever includes piperacillin/tazobactam administered after collecting sepsis screening samples, including blood cultures. Gentamicin is added based on clinical judgment, taking into account individual risk factors, past infections, and known resistance patterns. While the study provides important insights, larger multicenter studies are warranted to validate these findings and explore their broader applicability.
